# Two types of GLR channels cooperate differently in light and dark growth of Arabidopsis seedlings

**DOI:** 10.1186/s12870-023-04367-9

**Published:** 2023-07-14

**Authors:** Weronika Krzeszowiec, Aleksandra Lewandowska, Jan Jakub Lyczakowski, Kateryna Bebko, Sandra S. Scholz, Halina Gabryś

**Affiliations:** 1 Department of Plant Biotechnology, Faculty of Biochemistry, Biophysics and Biotechnology, Jagiellonian University in Kraków, Gronostajowa 7, Kraków, 30-387 Poland; 2 Matthias Schleiden Institute of Genetics, Bioinformatics and Molecular Botany, Department of Plant Physiology, Friedrich-Schiller-University Jena, 07743 Jena, Germany

**Keywords:** Arabidopsis, Aequorin, Blue light, CNQX, Darkness, GLR channels, MK-801, Photoreceptor mutants, Red light, Seedling growth

## Abstract

**Background:**

GLutamate Receptor-like (GLR) channels are multimeric, ionotropic, ligand-gated plant transmembrane receptors. They are homologous to mammalian glutamate receptors, iGLuRs, which are critical to neuronal function. GLRs have been reported several times to play a role in photomorphogenesis. However, to date, no study has looked at the mechanism of their involvement in this process. Here we focused on examining the impact of GLRs on the regulation of early seedling growth in blue light, red light, and in the dark.

**Results:**

Wild type and six photoreceptor mutant seedlings were grown on media supplemented with known iGLuR/GLR channel antagonists: MK-801, which non-competitively blocks NMDA channels in mammalian cells, and CNQX, known for competitive blocking of AMPA channels in mammalian cells. The lengths of hypocotyls and roots were measured in seedlings of *phyA*, *phyB*, *phot1*, *phot2*, *cry1*, and *cry2* mutants after 7 days of in vitro culture. Changes in growth parameters, both in light and in darkness upon application of chemical antagonists, show that both types of GLR channels, NMDA-like and AMPA-like, are involved in the regulation of seedling growth irrespective of light conditions. Analysis of seedling growth of photoreceptor mutants indicates that the channels are influenced by signaling from phot1, phot2, and cry1. To extend our analysis, we also evaluated the elicitation of a calcium wave, which is likely to be partially driven by GLRs, in Arabidopsis seedlings. The changes in cellobiose-induced calcium waves observed after applying GLR inhibitors suggest that both types of channels likely cooperate in shaping Arabidopsis seedling growth and development.

**Conclusions:**

Our work provides the first experimental evidence that two types of GLR channels function in plants: NMDA-like and AMPA-like. We also demonstrate that the channels are involved in seedling growth and development, at least partially through modulation of calcium signaling, but they are unlikely to play a major role in photomorphogenesis.

**Supplementary Information:**

The online version contains supplementary material available at 10.1186/s12870-023-04367-9.

## Background

GLutamate Receptor-like (GLR) channels are nonselective, ionotropic, ligand-gated receptors that form multimeric transmembrane structures in plant cells. They are homologous to ionotropic GLutamate Receptors (iGluRs) that play a key role in neurotransmission in the central nervous system of vertebrates. iGluRs are known for over 40 years (history review [1]), while the plant GLRs were discovered in 1998 [2]. Studies using chemical agents divided animal iGluRs into three groups: NMDA, AMPA, and kainate receptors. The receptors are named after the agonists that activate them: NMDA (N-methyl-d-aspartate), AMPA (α-amino-3-hydroxyl-5-methyl-4-isoxazole-propionate), and kainic acid. The newest information about similarities and differences between the plant and animal channels comes from X-ray crystallography and single-particle cryo-EM, which were used to solve the Arabidopsis GLR3.4 structure in a deactivated state [3]. On that basis GLRs have been predicted to share the structural domain organization with iGLuRs. The gating mechanism for both the animal iGLuRs and the plant GLRs depends, in principle, on the binding of glutamate and glycine to the multimer [3, 4].

There is a growing interest in GLRs mainly due to their role in vital physiological and developmental processes in plants. Among them are seed germination, pollen tube growth, reproduction and chemotaxis, regulation of stomatal aperture, ion transport, long-distance signaling, responses to salt stress, wounding, and pathogens (see reviews: [5, 6]). The abundance and diversity of processes listed above point to the necessity of studies which lead to a better understanding of the GLR channels contribution at functional and mechanistic levels. Another fact that supports the importance of GLRs in plant life is an increase in the number of GLR genes in plants compared to their counterparts, iGluRs in animals, where they play a fundamental role in signaling through the synapse. Since the channels participate in ion transport and affect calcium homeostasis, they can play a strategic role in environmental signal transmission, deemed to be of key importance also for plants. Their involvement in plant response to stresses, already documented for Arabidopsis [6], *S. lycopersicum* [7], and rice [8], represents an attractive topic, not only for the basic research, but also for agriculture.

Most of the above processes in which GLR channels participate are regulated by light. Light is a major stimulus that not only powers the energy metabolism of plants but also works as a signal which controls the growth of organs during ontogenesis. Light perception and signal transduction, via photoreceptors, are key to most stages of plant development, from germination through flowering and finally to senescence. With a few exceptions, the pathways for signal transduction from photoreceptors are known only fragmentarily.

One of the major physiological events in seedling growth is the inhibition of hypocotyl growth mediated by blue light. The early phase of suppression is phototropin1- dependent, while after about 30 min the cryptochrome-dependent phase begins [9, 10]. At least two events associated with ion fluxes occur during growth inhibition: (1) depolarization of the plasmalemma and (2) a transient increase of Ca^2+^ concentration in the cytosol [11]. Direct evidence of the participation of calcium ions in this process is the result of an experiment in which blocking the calcium wave with calcium chelator, BPTA, revoked the inhibition of hypocotyl growth mediated by *phot1*. The calcium channels involved have not been identified so far. One possibility is the function of the GLR channels in this process. In the first publication about GLRs in plants, Lam [2] showed that DNQX, an inhibitor of AMPA and kainite receptors, partly blocks the inhibition of hypocotyl growth by light in Arabidopsis seedlings. Similarly, seedlings treated with an agonist of iGluRs, BMAA, reduced light-induced shortening of hypocotyls [12]. Although these results seem contradictory, both give evidence in support of the participation of the GLR channels in the light control of seedling growth.

The present study was preceded by our research on light-driven *GLR* gene expression. We found expression of genes encoding seven (*GLR1.1*, *GLR2.7*, *GLR3.1*, *GLR3.2*, *GLR3.3*, *GLR3.5*, *GLR3.7*) out of 15 GLRs were found to be upregulated by a concerted action of photoreceptors in mature leaves of Arabidopsis [13]. This transcriptional upregulation was mainly regulated by the activity of phytochromes, which were assisted by cryptochromes. Only in the case of *GLR1.1*, we observed the contribution of phototropins to its transcriptional control. Knowing that light regulates the expression level of several *GLRs*, we wanted to evaluate if the resulting proteins also work in a light-controlled manner. Our study aims to answer the question of whether GLR channels specifically contribute to the light-directed growth and development of Arabidopsis at an early seedling stage. We used combined genetic, cell biological, and pharmacological approaches for that purpose.

## Results

### The influence of GLR inhibitors on seedlings growth

**Fig. 1:**
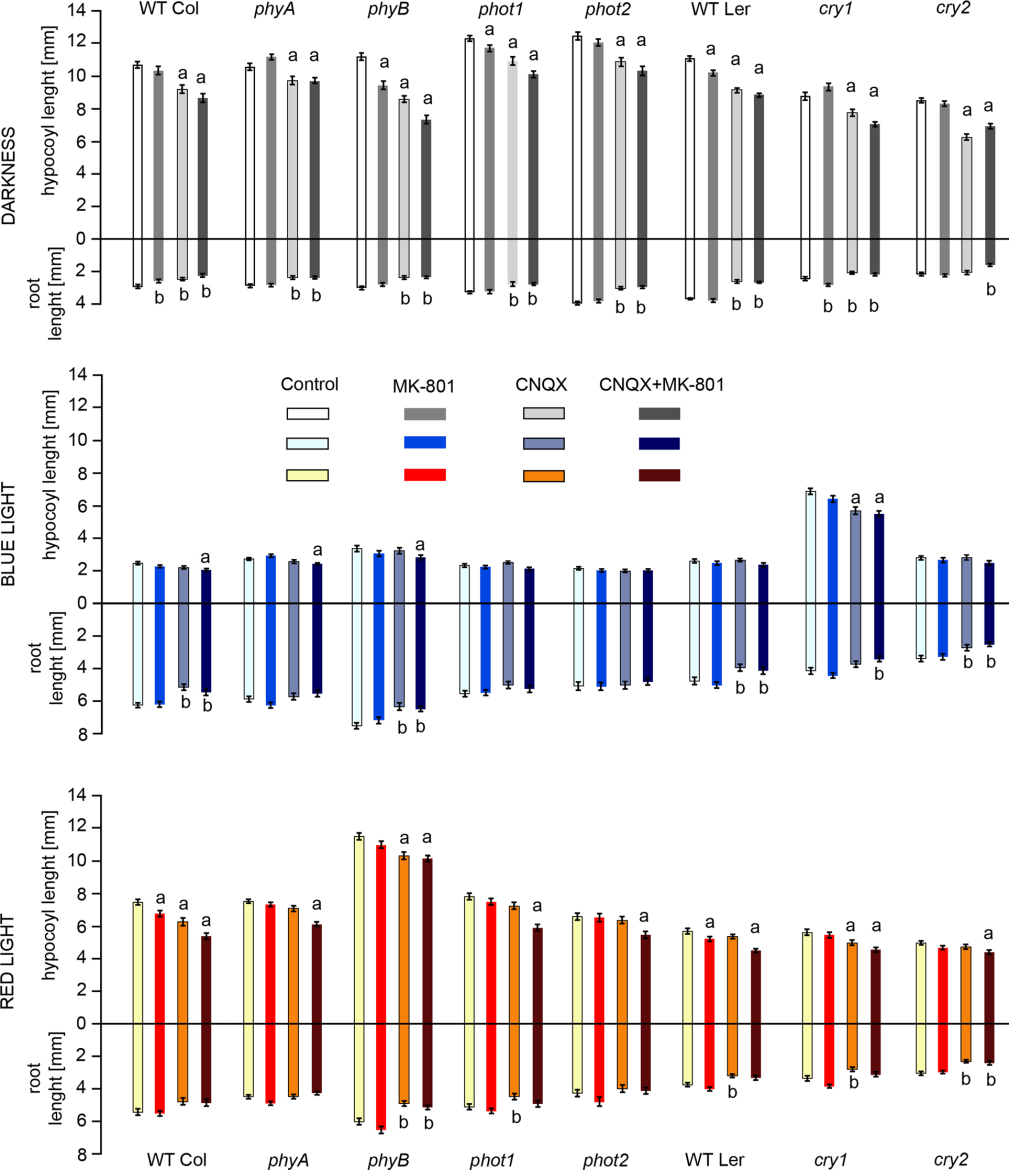
Average lengths of hypocotyls and roots visualized as complete seedlings of *Arabidopsis thaliana* Columbia (background for *phy* and *phot* mutants) and *Landsberg erecta* ecotypes (background for *cry* mutants). Zero indicates the position of the collars. The seedlings were grown in darkness (grey bars), blue light (shades of blue bars), and red light (shades of red bars). The growth medium was supplemented with 0.5 mM MK-801, 0.5 mM CNQX, or 0.5 mM (MK-801 + CNQX). Control seedlings grew on 1/2MS with 0.5% DMSO. The results collected in graphs represent means of three biological replicates with error bars denoting standard error of mean (SEM). Analysis by one-way ANOVA with Dunnett’s multiple comparisons test. a/b denote statistical differences for hypocotyl/root lengths between control and inhibitor-containing samples

To determine the involvement of GLRs in seedling growth we performed several experiments on *Arabidopsis thaliana* WT and mutant plants, grown in D (dark), BL (blue light), or RL (red light) on agar plates. Because the inhibitors could have different effects on the apical meristems of seedlings, we decided to measure hypocotyls and roots separately. Each plate contained WTs and mutants grown simultaneously. Figure 1 shows the average lengths of both organs assembled into a complete seedling. Additional file 1 demonstrates the results for each photoreceptor mutant separately.

In the WT plants, GLR inhibitors affected the elongation of both organs, with hypocotyls more susceptible than roots. The strongest inhibitory effect on elongation was seen in the presence of both inhibitors. The impact of MK-801, which non-competitively blocks NMDA channels in animal cells, was evident only in two cases: inhibition of root growth in D and inhibition of hypocotyl growth in RL. The second inhibitor, CNQX, known to block AMPA channels in a competitive manner in animal cells, inhibited root growth in D and BL, and hypocotyl growth in D and RL. The differences in the hypocotyl growth between the control and other samples are very small in BL.

The influence of GLR inhibitors on the early growth of photoreceptor mutants in D or under BL or RL is shown in Fig. 1. In control, inhibitor-free samples, the length of seedlings depended on light conditions. In darkness, the smallest seedlings were these of *cry1* and *cry2* mutants. The longest seedlings were these lacking phototropin photoreceptors. In BL, which arrests hypocotyl growth, *cry1* mutants were uninhibited and showed elongated phenotypes. Also, *phyA* and *phyB* mutants were slightly longer than WT plants. Moreover, roots in *phyB* mutants were particularly elongated. In RL, both organs of *phyB* seedlings were the longest.

All investigated photoreceptor mutants responded to GLR inhibitors similarly to WT plants. The exceptions are listed below. In darkness, MK-801 did not inhibit the growth of *phyA*, *phot2*, and *cry2* mutants. What is more, seedlings of *phyA* and *cry1* grew slightly longer in the presence of MK-801. In the case of *cry1* roots, this growth increase was significant when compared to the control. CNQX alone caused the shortening of both seedling organs in most genotypes analysed. This shortening was statistically significant in all cases except for the roots of *cry2* plants. It is worth pointing out that in *phyA* mutants the CNQX acted as strongly as both inhibitors together. In BL, the growth of *phot1* and *phot2* was unaffected by inhibitors. *phyB* and *cry2* mutants behaved like WT plants, Columbia and Landsberg erecta respectively, showing statistically significant inhibition of both, hypocotyl and root elongation. In other genotypes, the influence of inhibitors was smaller than in the dark but still visible. In RL phytochrome mutants responded to inhibitors qualitatively like in the BL but the extent of the response was stronger, whereas *phot1* and *phot2* were inhibited similarly as in the dark. *cry1* and *cry2* mutants were inhibited in the presence of CNQX and both inhibitors.

Overall, the effects of GLR inhibitors were most pronounced in the dark and attenuated in light conditions, particularly in BL. One important exception was the *cry1* mutant for which the hypocotyl growth under BL and RL conditions remained noticeably sensitive to GLR channel blocking.

**Fig. 2:**
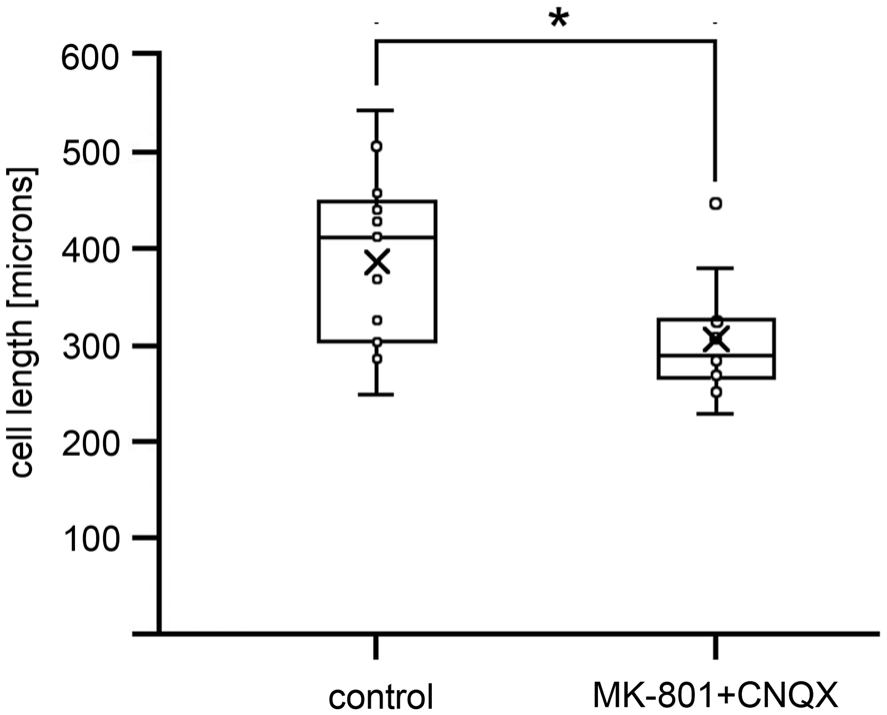
Cell lengths in etiolated hypocotyls of *Arabidopsis thaliana* (Col) in seedlings grown on ½ MS supplemented with 0.5% DMSO (control) and in the presence of inhibitors (0.5 mM MK-801 + CNQX). The number of cells measured 13. T-test analysis implies a significant difference in cell length, marked with an asterisk (P = 0.0109)

MK-801 and CNQX may reduce seedling growth via reducing cell elongation which is a prominent contributor to hypocotyl growth. To test this hypothesis we measured cell lengths in hypocotyls of etiolated seedlings, in control and MK-801 + CNQX treated WT samples (Fig. 2). Indeed cells in seedlings grown on the inhibitors were significantly shorter than in the control plants.

### Effects of various DMSO concentrations on seedling growth

**Fig. 3:**
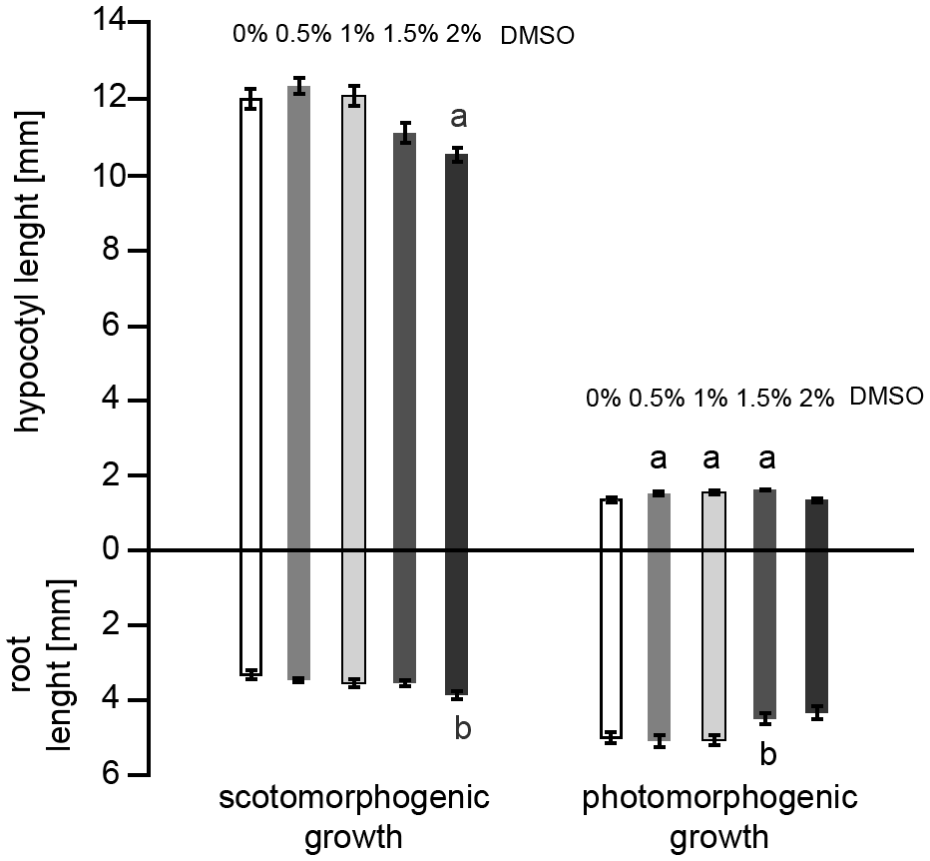
Skotomorphogenic and photomorphogenic seedling growth on media supplemented with various concentrations of DMSO. Average lengths of hypocotyls and roots visualized as complete seedlings of Arabidopsis thaliana (Col). Zero indicates the position of the collars. The seedlings were grown in darkness or white light of 120 µmol m^−2^ s^−1^. Analysis by one-way ANOVA with Dunnett’s multiple comparisons test. a/b denote statistical differences for hypocotyl/root lengths between control and DMSO-treated samples

To assess the possible effect of DMSO, the inhibitor solvent, on the seedling performance, we grew plants on a medium containing various DMSO concentrations and assessed hypocotyl and root growth (Fig. 3). In the dark, seedlings were insensitive to DMSO up to 1.0%. At higher concentrations, DMSO inhibits hypocotyl growth and promotes root growth. In white light, the seedlings were sensitive even to the lowest 0.5% concentration, with a small elongating effect on hypocotyls. Roots were insensitive up to 1.0% DMSO, at higher concentrations root elongation was inhibited.

### Effects of GLR inhibitors on cellobiose-induced calcium wave

**Fig. 4:**
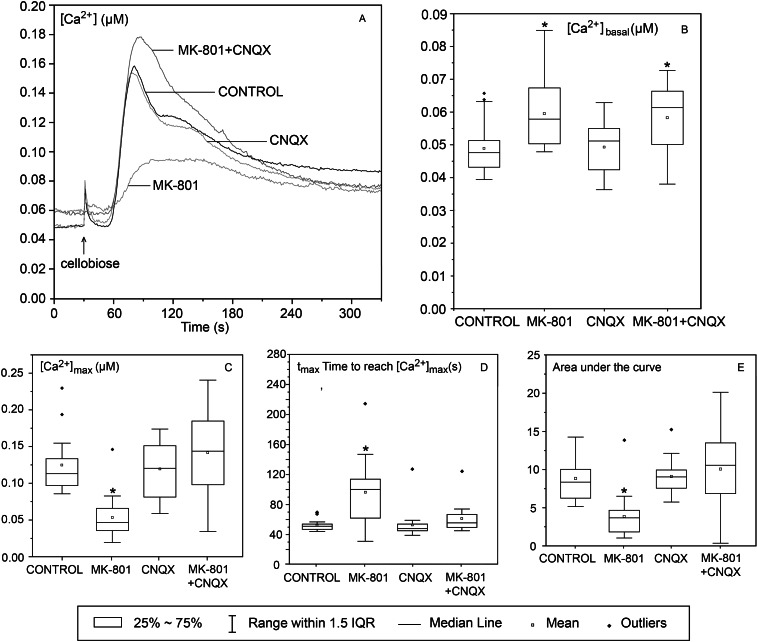
Effects of MK-801 and CNQX on cellobiose-induced calcium waves in light-grown Arabidopsis seedlings expressing cytosolic aequorin. The seedlings were incubated for 12 h with 0.5 mM MK-801, 0.5 mM CNQX, or both inhibitors. 0.5% DMSO was used as the control. Each curve represents average Ca^2+^ concentration changes from 16–18 independent experiments

Plant GLRs are known to be non-selective cation channels [5, 14]. We aimed to test the possibility that the observed growth alterations are due to the disturbance of calcium homeostasis. Therefore we tested how single and double inhibitors affect Ca^2+^ concentration in the cytoplasm. The experiments were conducted on light-grown Arabidopsis seedlings expressing cytosolic aequorin, a calcium-sensitive luminescent protein. We used cellobiose as the calcium wave elicitor [15]. The averages for 16–18 measurements are shown in Fig. 4.

The maximum Ca^2+^ concentrations were reached at 51, 107, 49, and 57 s (t_MAX_ average) after cellobiose addition for the control, MK-801, CNQX, and both inhibitors respectively. The long value of t_MAX_ average in the case of MK-801 treated seedlings is due to a large scatter of individual values. This caused a plateau on the lowest curve in Fig. 4A while the single curves had clear-cut maxima. Calcium waves in seedlings incubated with CNQX were very similar to those in control samples. MK-801 caused an increase in the basic Ca^2+^ concentration (Fig. 4. A and B) and a dramatic drop in the cellobiose-induced calcium wave (Fig. 4. A, C, and E). Unexpectedly, the calcium wave recorded in the presence of both MK-801 and CNQX was very similar to the control one. Thus the presence of CNQX counteracted the dramatic effect of MK-801. However, the basic level of cytoplasmic calcium was elevated, as in the presence of MK-801 alone (Fig. 4. A and B).

## Discussion

### Division of GLR channels

The classic division of animal iGLuR channels into AMPA, NMDA, and kainate subtypes is solely based on their pharmacological profile. There is a tendency to copy this division into plant GLR channels while no research-based evidence supporting the existence of both types of channels in plants can be found. Chiu and colleagues [16], based on phylogenetic analyses suggested that the divergence of animal iGluRs and plant GLR genes preceded the divergence of NMDA and AMPA subtypes. Here we have used single inhibitors to block only one subtype of GLR channels or two inhibitors of both GLR subtypes simultaneously. The strongest inhibitory effect on seedling growth was seen, almost in all cases, in the presence of both inhibitors. Thus, our results indicate the presence and interaction of both channel subclasses. This may suggest that both types of channels were present in the common ancestor of plants and animals.

### Involvement of GLR channels in the regulation of seedling growth

The first publication about GLR channels described a partial abolition of light-mediated growth inhibition of Arabidopsis seedlings by an AMPA channel inhibitor DNQX [2]. Since then, GLR channels have been repeatedly reported to play a role in photomorphogenesis. However, to date, no study has actually looked into the mechanism of their involvement in photomorphogenesis. Our research addressed this particular issue with a comprehensive experimental approach. We studied the impact of MK-801, CNQX, and both types of GLR antagonists on the growth of seedlings of *phyA*, *phyB*, *phot1*, *phot2*, *cry1*, and *cry2* mutants grown in the dark and under blue and red light. With this approach, we could identify GLR subtypes active in the signaling pathway of a particular photoreceptor.

The inhibition of seedling growth by GLR channel antagonists occurred in blue light, red light, and in darkness. Unexpectedly, GLR channels turned out to be most obviously involved during skotomorphogenesis. The overall pattern of inhibition by MK-801 and/or CNQX was much more limited in light-grown seedlings. In photoreceptor mutants, the growth responses of seedlings to inhibitors were comparable to those observed in wild-type plants. Only the growth of *phot1* and *phot2* seedlings cultivated in BL (which by itself exerts the strongest growth inhibition) was completely unaffected by GLR channel antagonists. At this moment, we cannot yet provide an explanation for this intriguing observation.

It is generally accepted that GLR channels play a role in root growth and development. Experimental evidence for that were presented for AtGLR3.2, AtGLR3.3, AtGLR3.4, and AtGLR3.6 [17–19], and for OsGLR3.1 in rice [20]. Other studies showed that glutamate, the main agonist of iGLuRs, is involved in root expansion in Arabidopsis e.g. [21, 22]. Our studies are consistent with these findings as we showed inhibition of root growth in the presence of GLR antagonists. In our experimental system, roots were more sensitive to CNQX than to MK-801. This suggests that AMPA-like subtypes of GLRs are more important in root growth. The strongest inhibition was visible for both antagonists. MK-801, occasionally stimulated elongation of the roots, with the statistical significance in *cry1* mutant in darkness. Thus, both subtypes of GLR channels appear to cooperate in the regulation of root growth.

The involvement of GLR channels is mostly visible in roots grown in darkness. However, it cannot be excluded that by illuminating whole seedlings in the adopted experimental setup, we interfered with the normal photomorphogenic growth of roots which, in natural conditions, grow in the soil, in darkness.

Hypocotyl growth is regulated mainly by light. In the dark hypocotyls are elongated. In red light, their length is partially reduced whereas in blue light it is strongly reduced. These classical effects are clear also in our experiments. As in roots, the most pronounced inhibitory effect of MK-801 and CNQX was visible in darkness, in the presence of both inhibitors. Only, in the *cry1* mutant, was the inhibitory effect similar in D, BL, and RL.

The results of our study indicate that an indirect relationship exists between BL photoreceptors and the GLR channels in the regulation of seedling growth. Firstly, this is reflected in the insensitivity of phototropin mutants to GLR antagonists. Secondly, in *cry1* no growth reduction is observed in BL, and, unlike in any other genotype analyzed, MK-801 and CNQX appear to still be active in inhibiting hypocotyl and root elongation. The nature of these relationships and the role of GLR channels in growth require further studies to add more details to the mechanism of early plant growth.

**Fig. 5:**
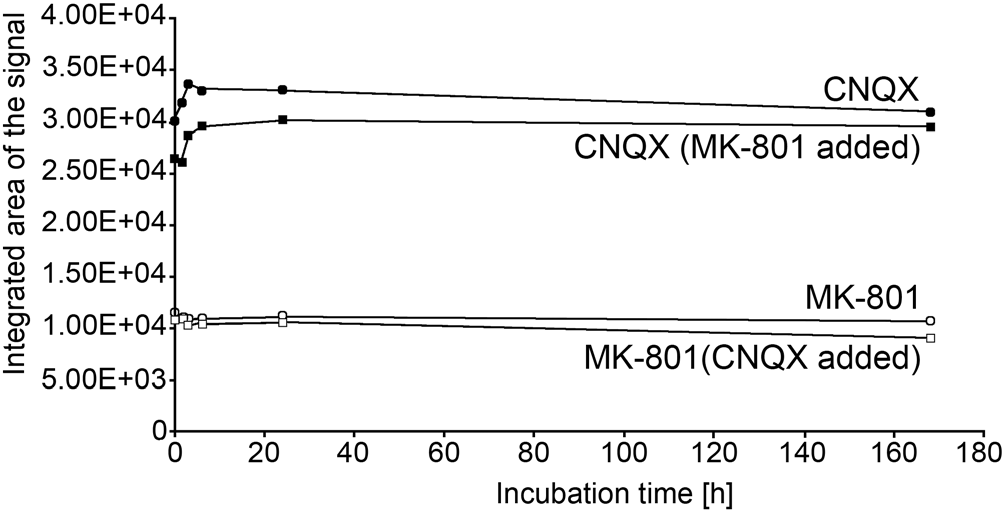
Integrated area of MK-801 and CNQX HPLC signals measured over time for samples taken from an aqueous solution stored at room temperature. The peak area for both inhibitors remains stable, suggesting that no MK-801 and CNQX degradation is happening during the course of the experiment

Our results differ in some aspects, from these of Lam [2]. In this study Arabidopsis seedlings growing in white light in the presence of an AMPA channel antagonist were more elongated compared to the control; the antagonist was active only in the light conditions. In our experiments, the seedlings were almost always shortened by an analogous antagonist, irrespective of light conditions (R, B, or D); the most pronounced inhibitory activity was detected in the dark-growing seedlings. Also, contrary to the commented work, we did not notice any obvious differences in greening of the antagonist-treated vs. control seedlings. The discrepancies between the results may be caused by using different AMPA inhibitors: DNQX [2] and CNQX (current study). Another potential source of discrepancies may be connected with the use of DMSO as MK-801/CNQX solvent. While commonly used GLR channel inhibitors are solubilized in DMSO, this compound was shown to interfere with physiological processes in rice [23]. We examined the effect of DMSO in a systematic way, and found out that it affected Arabidopsis seedling growth starting from concentrations necessary to effectively dissolve the hydrophobic inhibitors (Fig. 4). Hypocotyls were found to be more sensitive to DMSO than roots. Intriguingly, in white light, DMSO was affecting the seedling growth stronger than in the dark. For example, in the range of 0,5% -1,5% DMSO slightly but significantly counteracted the inhibitory effect of light on hypocotyl elongation. Thus, discrepancies between results presented in the literature may be due to different solvent concentrations of the inhibitors. We suggest that concentrations of DMSO higher than 0.5% should not be used in plant pharmacological studies using this solvent. We also observed that the longer the seedling grows in the presence of DMSO, the greater the side effects. We also tested whether the inhibitors could be degraded in a growth experiment carried out for seven days, including five days at room temperature. In studies using HPLC, we confirmed the stability of the inhibitors in aqueous solutions (Fig. 5). Thus, we believe that the effect of inhibitors during seedling growth was continuous throughout the experiment.

### Effect of inhibition of GLR channels on Ca^2+^ waves

To link the action of channel inhibitors with an expected disturbance of the calcium wave, we performed cytosolic Ca^2+^ concentration tests using transgenic WT Arabidopsis expressing aequorin. The results were rather unexpected. In the presence of MK-801, the calcium wave was significantly reduced and deformed. Both the increase of calcium concentration and its pumping out of the cytoplasm were much slower than in the control. On the contrary, CNQX alone did not affect the calcium wave. Animal studies showed the presence of two subtypes of AMPA channels, one permeable and the second non-permeable for Ca^2+^ [24]. Thus, the AMPA-like channels in Arabidopsis seedlings may serve to regulate the transport of other ions, for example, K^+^ or Na^+^ [25]. The recovery of the calcium wave following the concomitant application of both inhibitors points to an interaction between AMPA and NMDA channels, that has not been postulated in plants to date. If the inhibitors affected independent channels, one would have expected a reduction of the calcium wave similar to that observed for MK-801 only. Our results show that the regulatory input of GLR channels on seedling growth may involve control of ion transport by both types of channels. However, elucidation of the role played by that control in growth regulation requires numerous independent studies. So far only very scarce data is available on the composition of GLR channels in vivo, modes of their regulation (e.g. agonists), and their possible interactions with other cellular components (proteins, phosphoinositides, etc.). These facts must be established to develop a reasonable model of GLRs functioning in plants.

## Conclusions

We have designed a double-inhibitor strategy to address the question about GLR channels participation in seedling growth in Arabidopsis. Our results show that two types of GLR channels are present in plants, NMDA-like and AMPA-like.

GLR channels are involved in the growth response of seedlings to darkness and light absorbed by phototropins and cryptochrome. Overall, dark growth is affected stronger than light growth by antagonists of GLR channels. This indicates that the channels may not play a major role in photomorphogenesis.

In addition, we clarify two methodological issues related to the GLR studies, including, the stability of inhibitors MK-801 and CNQX in aqueous solutions as well as the possible effect of DMSO, a standard solvent of the inhibitors, on the studied processes.

Additionally, we show the effect of inhibitors on the cellobiose-induced calcium waves. We anticipate that further studies on explaining observed effects will provide insights into the mechanism of action of GLR channels in plants.

Studying the role of GLRs in plants is important for determining signaling pathways that lead to growth and development, adaptation to environmental cues, and response to pathogens. We believe that our work will help to design new experiments by taking into account the presence of both types of channels in plants.

## Methods

### Plant material

*Arabidopsis thaliana* WT (wild type) and mutant seeds were submerged in distilled water with a drop of household detergent for 10 min and sterilized for 5 min in 20% bleach (ACE). Then, they were rinsed at least four times in sterile water. Seedlings were grown on agar-covered square plates in a vertical position.

Seeds of *Arabidopsis thaliana* wild type Col-0 (ID: N60000) and Landsberg erecta (ID: NW20) were purchased from The Nottingham Arabidopsis Stock Centre (NASC). We used the following photoreceptor mutants: *phyA-211* [26], *phyB9* [27], *phot1* (SALK_088841C, NASC), *phot2* [28], *cry* [29], and *cry2* [30]. Seeds of cry mutants were a gift from Professor Chentao Lin (University of California, Los Angeles, CA, USA). As the available cryptochrome mutants had been obtained in Landsberg erecta background, the results for *cry1* and *cry2* were compared to WT of that ecotype.

The Arabidopsis transgenic line expressing cytosolic aequorin (a calcium-sensitive luminescent protein) was used to assess the cytosolic calcium [31]. The seeds were a kind gift from Professor Marc R. Knight (Department of Biosciences, Durham University, UK).

### Preparation of inhibitor-containing media

The agar medium was calibrated to pH of 5.75. It contained ½ MS (Sigma Aldrich), 0.8% Agar (BTL sp. z o. o.) and selected GLR inhibitors. The concentrations of inhibitors used were 0.5 mM MK-801 (Sigma Aldrich), 0.5 mM CNQX (Sigma Aldrich), 0.5 MK-801 + 0.5 mM CNQX, and 0.5% DMSO (Sigma Aldrich) as a control. Since the inhibitors are weakly soluble in water, stock solutions were prepared in DMSO at the concentration of 90 mM for each inhibitor. At their final concentrations, the concentration of DMSO was 0.5%. The preparation of the CNQX stock solution required heating at about 37 °C and vigorous mixing. The inhibitors were added from 90mM stocks to the liquid but cooled agar medium in a Falcon tube. The medium was immediately mixed and poured onto plates.

### Growth conditions

Plants were sown under sterile conditions and incubated in darkness in a cold room at 4 °C for 3 days. Germination was induced by irradiation of seeds for 2 h with white light of about 100 ± 20 µmol m^− 2^ s^− 1^ in a growth chamber (Sanyo MLR-350 H, fluorescent lamps Philips Master TL-D-36 W/840, Osram L36 W/77 Fluora, Activa 172 − 36 W, Sylvania Gro-Lux F36W/GRO-T8) at 23 ± 2 °C, 80% relative humidity. The induction was done after the cold treatment, from 12 to 2 pm for all subsequent light regimes.

Following the induction of germination, plants were grown in short days 10 h L/14 h D in color lights or darkness (D). Blue light (BL) was obtained from LXHL-PR09 LEDs (Ledium Ltd. Hungary) with a maximum emission at 455 ± 20 nm (half-band width). Red light (RL) was obtained from Luxeon Rebel ES LEDs (Philips Lumileds Lighting Comp.) with a maximum emission at 655 ± 14 nm. Seedlings were grown for 5 days. Photographs (Lumix, Panasonic DMC-FZ1000) of the plates were taken on the 5th day around 11 am to 12 noon. The whole experiment lasted one week.

### Seedlings length measurements

Lengths of seedlings were determined on photographs using ImageJ software. Each seedling was measured from the shoot apical meristem to the root tip and then the hypocotyl was measured from the shoot apical meristem to the root collar. At least three biological replicates were done for each experiment. In total over 10,000 seedlings were measured. Mutant and WT plants were grown simultaneously. The BL and RL experiments were also done simultaneously, in the same dark room. Special screens were used to make sure that no reflections reach seedlings growing in a different light. The numbers of the assessed seedlings are given in the supporting information Additional file 2.

### Influence of DMSO on seedling growth

Additional experiments were carried out to assess the possible impact of DMSO, used to dissolve inhibitors, on seedling growth. For these experiments, seedlings were grown on plates with ½ MS supplemented with increasing concentrations of DMSO: 0%, 0.5%, 1%, 1.5%, and 2%, in the dark or white light. After sterilization, sowing of seeds, and 3 days of cold treatment at 4 °C, the germination was induced in a Sanyo growth chamber, as described in the Growth Conditions section. Subsequently, half of the plates were wrapped in aluminum foil for skotomorphogenic experiments. The remaining plants were grown for 5 days in the same growth chamber, in white light of 100 ± 20 µmol m^− 2^ s^− 1^.

### Stability of CNQX and MK-801 in aqueous solutions

In our experiments, plants were incubated with GLR inhibitor solutions on plates over a total of one week. Therefore we wanted to make sure that the inhibitors remain stable and active for the duration of the experiment. To evaluate that we stored aqueous solutions of MK-801 (0.5 mM), NQX (0.5 mM), and MK-801+ CNQX (both at 0.5 mM) at room temperature in white light for one week and took samples at specific time points to evaluate the concentration of inhibitors. Inhibitor concentration was evaluated by separating the solution in an Agilent Technologies 1260 Infinity II HPLC system using a Nucleosil 100 C18 5 μm, 25 × 0,4 mm column (Teknokroma). Flow protocol for mobile phase used is detailed in Table 1. Flow was set to 1 mL/min. Area of signal specific for MK-801 and CNQX was determined for each timepoint and solution composition and plotted against time (Fig. 5). No significant degradation of the inhibitors was detected in the experiment.

**Table 1:** Solvents use as a mobile phase in specific parts of the separation protocol

Time (min)	Water (%)	Acetonitrile:methanol:water 72:18:10 v/v (%)	Methanol (%)
0	85	15	-
10	-	100	-
15	-	100	-
16	-	-	100
21	-	-	100
22	85	15	-
25	85	15	-

### Cytosolic calcium evaluation

Calcium concentration in the cytosol was measured using a luminometric method described previously [32]. Arabidopsis 7-day-old seedlings expressing cytosolic aequorin (a calcium-sensitive luminescent protein) were dark-incubated for 12 h with a freshly prepared 7.5mM coelenterazine (p.j.k) and GLR inhibitors in 1.5ml Eppendorf tube. The same concentrations of inhibitors and the control were used as described in Methods, in the “Preparation of inhibitor-containing media” section. Aequorin luminescence emitted from the seedlings was measured using a GloMax 20/20 luminometer (Promega) working with the software GLOMAX SIS v1.10.0. After incubation, each tube was placed in a tube holder of the luminometer in total darkness. The basal luminescence was recorded for 30 s. 1mM cellobiose (Merck) was used as an elicitor to induce a transient calcium wave. The calcium wave was measured for 5 min. Finally, the remaining reconstituted aequorin was discharged by adding 1 M CaCl_2_ in 10% ethanol. The luminescence counts obtained with the luminometer were calibrated into Ca^2+^ concentrations using the equation of Rentel and Knight [31].

## Electronic supplementary material

Below is the link to the electronic supplementary material.

Supplementary Material 1

## Data Availability

The data that support the findings of this study are available on request from the corresponding author, [WK], upon reasonable request.

## Abbreviations

BL: Blue light
D: Darkness
RL: Red light
WT: Wild type
