# Supplementary material for: Two types of GLR channels cooperate differently in light and dark growth of Arabidopsis seedlings
Source: BMC Plant Biol. 2023 Jul 14;23:358. doi: 10.1186/s12870-023-04367-9 (PMC10347744; doi:10.1186/s12870-023-04367-9)
Supplement: Supplementary file 1 — Supplementary Material 1 [file 12870_2023_4367_MOESM1_ESM.pdf]

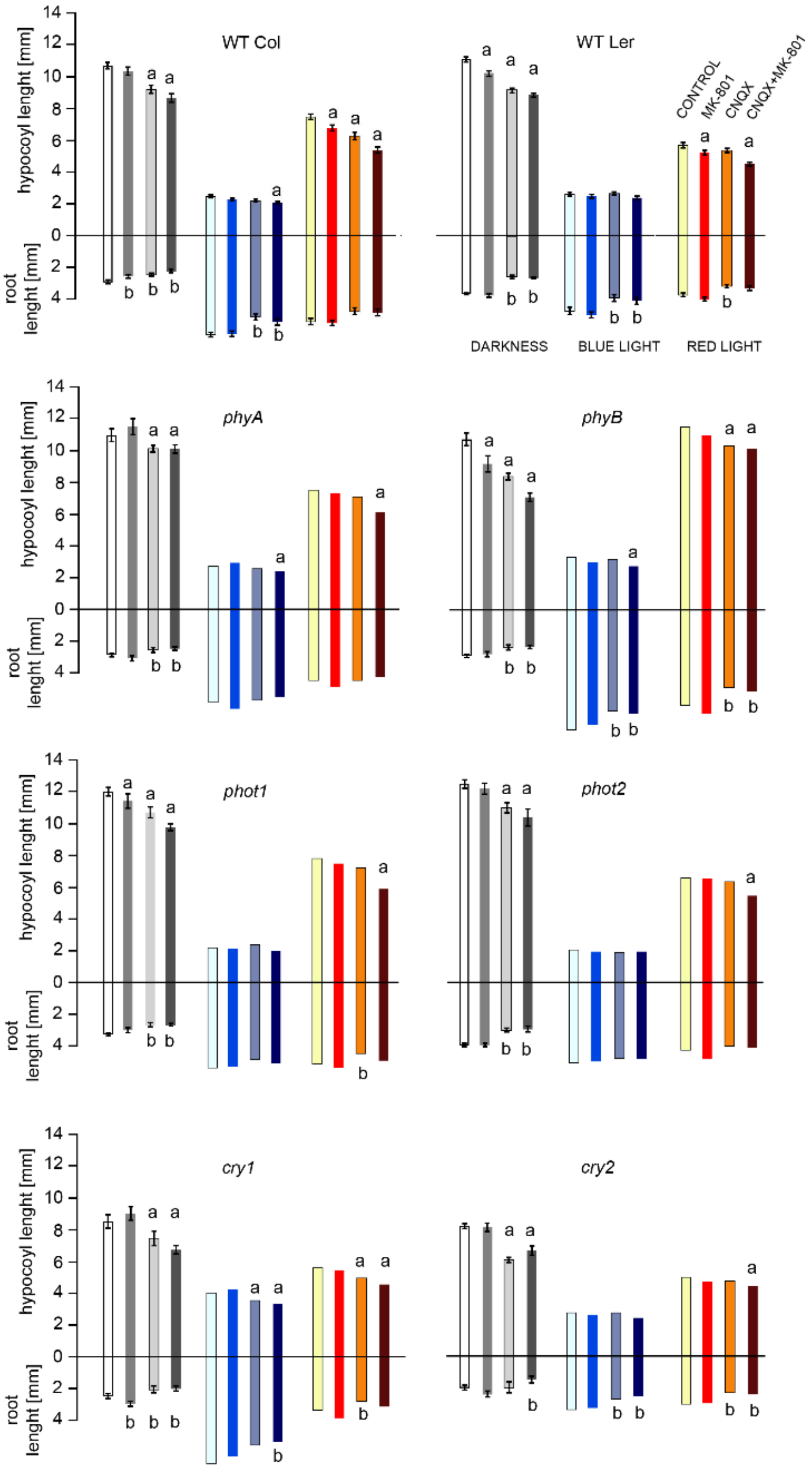

Additional file 1. Average lengths of hypocotyls and roots visualized as complete seedlings of *Arabidopsis thaliana* Columbia (background for *phy* and *phot* mutants) and Landsberg erecta ecotypes (background for *cry* mutants). Zero indicates the position of the collars. The seedlings were grown in darkness (grey bars), blue light (shades of blue bars), and red light (shades of red bars). The growth medium was supplemented with 0.5 mM MK-801, 0.5 mM CNQX, or 0.5 mM (MK-801+CNQX). Control seedlings grew on 1/2MS with 0.5% DMSO. The results collected in graphs represent means of three biological replicates with error bars denoting standard error of mean (SEM). Analysis by one-way ANOVA with Dunnett's multiple comparisons test. a/b denote statistical differences for hypocotyl/root lengths between control and inhibitor-containing samples

Additional file 2. The total number of seedlings measured in the experiments. Sample size = a number of seedlings measured in the experiment. Control – growth medium ½ MS with 0.5% DMSO, MK-801 – ½ MS with 0.5 mM MK-801, CNQX – ½ MS with 0.5 mM CNQX, MK-801 and CNQX – ½ MS with 0.5 mM MK-801 and 0.5 mM CNQX

**DARKNESS** sample size	Ath WT Col	Ath *phyA*	Ath *phyB*	Ath *phot1*	Ath *phot2*	Ath WT Ler	Ath *cry1*	Ath *cry2*
control	93	67	89	99	106	205	135	105
MK-801	101	60	83	110	126	215	121	103
CNQX	101	65	105	108	110	192	127	110
CNQX and MK-801	109	65	87	108	116	200	131	101

**RED LIGHT** sample size	Ath WT Col	Ath *phyA*	Ath *phyB*	Ath *phot1*	Ath *phot2*	Ath WT Ler	Ath *cry1*	Ath *cry2*
control	78	79	82	77	67	77	74	85
MK-801	69	75	82	79	65	92	93	63
CNQX	81	73	79	83	46	87	92	77
CNQX and MK-801	79	78	79	71	57	84	97	76

**BLUE LIGHT** sample size	Ath WT Col	Ath *phyA*	Ath *phyB*	Ath *phot1*	Ath *phot2*	Ath WT Ler	Ath *cry1*	Ath *cry2*
control	92	88	79	74	56	83	96	72
MK-801	82	82	84	77	72	91	87	50
CNQX	88	86	86	81	70	95	96	76
CNQX and MK-801	81	90	82	77	71	85	82	66

**DMSO** sample size	0%	0.5%	1%	1.5%	2%
DARKNESS	163	159	164	121	159
WHITE LIGHT	147	141	147	117	32
